# The structure of blood–tumor barrier and distribution of chemotherapeutic drugs in non-small cell lung cancer brain metastases

**DOI:** 10.1186/s12935-021-02263-6

**Published:** 2021-10-24

**Authors:** Ling-yun Ye, Li-xiang Sun, Xiu-hua Zhong, Xue-song Chen, Song Hu, Rong-rong Xu, Xiao-ning Zeng, Wei-ping Xie, Hui Kong

**Affiliations:** 1 Department of Medical Oncology, Shanghai Pulmonary Hospital, Tongji University Medical School Cancer Institute, Tongji University School of Medicine, No. 507 Zhengmin Road, Shanghai, 200433 People’s Republic of China; 2 Department of Respiratory Medicine, The Affiliated Hospital of Xuzhou Medical University, No. 99 West Huaihai Road, Xuzhou, 221006 Jiangsu People’s Republic of China; 3 Department of Pulmonary and Critical Care Medicine, The Affiliated Wuxi People’s Hospital of Nanjing Medical University, 299 Qingyang Road, Wuxi, 214000 Jiangsu People’s Republic of China; 4 Department of Respiratory and Critical Care Medicine, The First Affiliated Hospital of Nanjing Medical University, Nanjing, 210029 Jiangsu People’s Republic of China; 5 Department of Respiratory Medicine, The First People’s Hospital of Changzhou, The Third Affiliated Hospital of Soochow University, No. 185 Juqian road, Changzhou, 213000 Jiangsu People’s Republic of China; 6 Department of Respiratory Medicine, Zhongda Hospital, Southeast University, Nanjing, 210009 Jiangsu People’s Republic of China

**Keywords:** Non-small cell lung cancer, Brain metastasis, Blood–brain barrier, Blood–tumor barrier, Chemotherapeutic drugs

## Abstract

**Background:**

Brain metastasis is an important cause of increased mortality in patients with non-small cell lung cancer (NSCLC). In brain metastasis, the blood–brain barrier (BBB) is frequently impaired, forming blood–tumor barrier (BTB). The efficacy of chemotherapy is usually very poor. However, the characteristics of BTB and the impacts of BTB on chemotherapeutic drug delivery remain unclear. The present study investigated the structure of BTB, as well as the distribution of routine clinical chemotherapeutic drugs in both brain and peripheral tumors.

**Methods:**

Bioluminescent image was used to monitor the tumor load after intracranial injection of lung cancer Lewis cells in mice. The permeability of BBB and BTB was measured by fluorescent tracers of evans blue and fluorescein sodium. Transmission electron microscopy (TEM), immunohistochemistry and immunofluorescence were performed to analyze structural differences between BBB and BTB. The concentrations of chemotherapeutic drugs (gemcitabine, paclitaxel and pemetrexed) in tissues were assayed by liquid chromatography with tandem mass spectrometry (LC-MS/MS).

**Results:**

Brain metastases exhibited increased BTB permeability compared with normal BBB detected by fluorescence tracers. TEM showed abnormal blood vessels, damaged endothelial cells, thick basement membranes, impaired intercellular endothelial tight junctions, as well as increased fenestrae and pinocytotic vesicles in metastatic lesions. Immunohistochemistry and immunofluorescence revealed that astrocytes were distributed surrounded the blood vessels both in normal brain and the tumor border, but no astrocytes were found in the inner metastatic lesions. By LC-MS/MS analysis, gemcitabine showed higher permeability in brain metastases.

**Conclusions:**

Brain metastases of lung cancer disrupted the structure of BBB, and this disruption was heterogeneous. Chemotherapeutic drugs can cross the BTB of brain metastases of lung cancer but have difficulty crossing the normal BBB. Among the three commonly used chemotherapy drugs, gemcitabine has the highest distribution in brain metastases. The permeability of chemotherapeutic agents is related to their molecular weight and liposolubility.

## Introduction

Lung cancer is one of the most common malignant tumors and remains the leading cause of cancer-related lethality worldwide [1, 2]. Non-small cell lung cancer (NSCLC), as the main histological type of lung cancer, accounts for 80% of all cases. Advanced imaging technologies, improved treatment approaches, and earlier detection of clinically silent lesions have prolonged the survival of patients with lung cancer [3]. However, one unfortunate consequence of prolonged survival is that patients may eventually suffer from tumor metastasis, especially brain metastasis.

Most metastatic brain tumors, accounting for about 45%, originate from lung cancer [4, 5]. Currently, patients with brain metastases are usually considered to be terminal with poor prognosis. Approximately 40–50% of patients diagnosed with NSCLC are estimated to develop brain metastasis during the course of the disease [6–8]. The overall survival for the patients is 2 months with palliative treatment, even with radiation therapy, the survival remains poor with median survival time of 7.6 months [6–8]. Thus, effective treatments of NSCLC with brain metastases are critical for improving the prognosis of advanced lung cancer patients.

The locations of brain metastases are related to blood flow and tissue volume, with approximately 80% detected in the cerebral hemispheres, 15% in the cerebellum, and 5% in the brainstem [3]. Despite of many advances that have been made in the treatment of NSCLC, including chemotherapy, surgery, targeted therapy, stereotactic radiosurgery (SRS), and whole-brain radiotherapy (WBRT), the prognosis of patients with brain metastases is still dismal [9–11]. Systemic chemotherapies have little success in the treatment of brain metastases which is at least partially due to the presence of blood–brain barrier (BBB) [12, 13]. The BBB is formed by specialized endothelial cells, pericytes and astrocytic perivascular endfeet. The adjacent cells are tightly connected to each other via intercellular junctions, preventing most molecules to pass through endothelial cells. In addition, the constituents of the BBB express high levels of active efflux drug transporters, such as the P-glycoprotein and multidrug resistance proteins [14]. Together, these factors curb the accessibility and delivery of therapeutic agents to the brain parenchyma. Some previous studies suggested that the BBB is disrupted in brain metastases, creating a blood–tumor barrier (BTB) [15]. It has been debated for several years whether BTB overrides BBB in metastases. In addition, the impact of BTB on chemotherapeutic drugs distribution remains unclear in brain metastases treatment [16, 17].

The present study aimed to investigate the structure and characteristics of the BTB in brain metastases of NSCLC. Using stereotactic intracranial injection of tumor cells in mice to establish brain metastasis model of lung cancer, the structural differences between healthy BBB and barrier of brain metastases were investigated. To provide the basis for choosing clinical chemotherapeutic drugs and optimizing treatment, we explored the distribution of routine chemotherapeutic drugs for NSCLC in brain metastases.

## Materials and methods

### Cell culture and labeling

Lewis cells were purchased from the Institute of Biochemistry and Cell Biology of the Chinese Academy of Sciences (Shanghai, China). The cells were propagated in RPMI 1640 medium (Gibco BRL) supplemented with 10% fetal bovine serum (ScienCell, USA), 100 U/ml penicillin and 100 U/ml streptomycin at 37 °C in a 5% CO_2_ humidified atmosphere. Luc-lentivirus was purchased from Genechem Co., Ltd. 2 × 10^4^ Lewis cells were propagated in 25 cm^2^ culture flask. After 24 h, the culture medium was changed, and then Luc-lentivirus was added to the flask according to the instructions (MOI = 20). After 96 h of continuous culturing, the cells were digested and subcultured. Stable cells were screened by puromycin (8 μg/ml). Then the screened cells were inoculated into 96 well plates. After the cells completely adhered to the well, 150 μg/ml d-fluorescein potassium salt (Shanghai Sciencelight Biology Science & Technology Co., Ltd.) was added and incubated at room temperature in the dark for 15 min. The cells transfection condition was detected by a fluorescence imaging system (an IVIS 200 system). When the fluorescence intensity of average single cell reached more than 10^2^ p/sec/cm^2^/Sr, it suggested that the cell transfection could meet the requirement of subsequent experiments.

### Animal experiments

Athymic male nu/nu mice (7–8 weeks of age, 21–25 g each) were purchased from Shanghai, China. The mice were maintained under controlled conditions (room temperature and 12-h light/dark cycle) and had access to standard food and water ad libitum. All experiments were conducted according to the guidelines of the National Institutes of Health. The experimental procedures were approved by the Institutional Animal Care and Use Committee of Nanjing Medical University. We used stereotactic intracranial injection of tumor cells to construct lung cancer brain metastasis model to study the permeability and structure of blood–brain barrier. In the experiment of studying the distribution of chemotherapeutic drugs, we used stereotactic intracranial injection and subcutaneous injection tumor cells to observe the distribution of chemotherapeutic drugs in brain and peripheral tumors.

### Stereotactic intracranial injection of tumor cells

Mice were anesthetized with 4% chloral hydrate and placed in a stereotactic frame (RWD Life Science, Shenzhen, China). A middle incision was made, followed by a 0.5-mm burr hole that was 2 mm lateral and 2 mm posterior to the bregma. Then, 4 µl of cell suspension containing approximately 5 × 10^4^ Lewis cells was slowly injected intracranially using a 10 µl microinjector syringe at 3 mm below the skull surface over a period of 3 min. The needle was then removed over a 3-min period. The burr hole was sealed with bone wax and the scalp sutured. The mice were monitored until regaining consciousness and were returned to their cages.

### Subcutaneous injection of tumor cells

After the nude mice were anesthetized with 4% chloral hydrate, the right ventral proximal axillary skin was disinfected with 75% alcohol, the cell density was adjusted to 10^9^/L, and 100 μl cell suspension was injected subcutaneously into the right ventral proximal axillary.

### In vivo bioluminescent imaging

Mice were i.p. injected with 150 mg/kg D-luciferin potassium salt [15 mg/ml in phosphate-buffered saline (PBS)] and anesthetized with isoflurane (2–3%). Ten minutes after injection, images were acquired using an IVIS 200 system. The bioluminescent signal was measured as photons per second per square centimeter in regions of interest using Living Image software.

### Intravascular injection and detection of fluorescence tracers

A solution of 2% Evans blue (EB, 4 ml/kg, Sigma, USA) was injected into each animal via the femoral vein and circulated for 30 min. After 25 min of EB circulation, fluorescein sodium (F-Na, 300 mg/kg, Sigma, USA) was administered intravenously to circulate for 5 min. Anesthetized mice were perfused with saline to wash out excess fluorescence tracers, and then euthanized by cervical dislocation, after which the brains were quickly removed and embedded in optimum cutting temperature (OCT, Sakura, USA) cryostat-embedding compound. Next, the brains were frozen in liquid nitrogen and sliced into 40-µm thick sections at − 19 °C. The penetration of fluorescence tracers through the BBB was further assessed by fluorescence microscopy (Leica, Germany).

### Hematoxylin and eosin staining

To confirm the presence of intracranial xenografts, anesthetized mice euthanized by cervical dislocation and whole brains of mice were removed and fixed in a neutral-buffered 10% formaldehyde solution overnight. Then, the brains were embedded in paraffin, sectioned into 4 μm slices and stained with hematoxylin and eosin (H&E).

### Transmission electron microscopy analysis

On the sixth day after stereotactic intracranial injection of Lewis cells, mice were anesthetized by intraperitoneal injection of pentobarbital sodium (70 mg/kg) and perfused through the left ventricle with 100 ml saline followed by 100 ml 4% paraformaldehyde. The brains were removed, fixed in glutaraldehyde, dehydrated in acetone, embedded with epoxy resin, and sliced into ultrathin sections. Then, the sections were observed under a transmission electron microscope (JEM-1010, Japan).

## Immunohistochemistry (IHC) method

Mice were euthanized as mentioned above, the brains were removed for alcohol dehydration and paraffin embedding. Paraffin-embedded tissue slices were deparaffinized in xylene, rehydrated in graded ethanol, and rinsed with PBS. Then, the slices were placed in a repair box filled with EDTA antigen repair buffer (pH 8.0) and incubated with BSA. The sections were then incubated overnight at 4 °C with polyclonal antibodies against GFAP (Abcam, USA) and CD31 (Santa Cruz, USA). After washing, the slides were incubated with secondary antibody (Life, USA) for 1 h at room temperature. Finally, the slides were visualized by incubation with 3,3′-diaminobenzidine (DAB) and counterstained with hematoxylin (37%).

### Immunofluorescence (IF) method

The procedure was like that used for IHC analysis. Briefly, paraffin-embedded tissue slices were deparaffinized in xylene, rehydrated in graded ethanol, rinsed with PBS, and incubated with primary and secondary antibodies. Then, the slices were incubated with Hoechst staining buffer for 30 min at room temperature and analyzed via fluorescence microscopy (Leica, Germany).

### Drug treatments

The chemotherapeutic drugs were administered 6 days after Lewis cells implantation in mice. All drugs were prepared immediately before use and were given at a dose volume of 10 ml/kg via tail vein. Paclitaxel was administered as an alcohol solution of Cremophor (Cremophor dose, 1.2 ml/kg), and gemcitabine and pemetrexed were given as a water solution. The drugs were administered at the dose level of 30 mg/kg to mice bearing tumors (n = 5 per group).

### LC-MS/MS analysis

As mentioned above, the mice in each group were anesthetized and then euthanized by cervical dislocation. Blood, brain tumors, healthy brain tissues and subcutaneous tumors were rapidly obtained at 15 min, 30 min, 1, 2 and 4 h after injection of the chemotherapeutic drugs for liquid chromatography-mass spectrometry analysis. Brain tumors were obtained as described above. Blood was taken by removing eyeballs of mice. Healthy mouse was perfused through the left ventricle after anesthesia, and the skull was cut to obtain healthy brain tissue. The axillary skin of mice was cut, and the subcutaneous tissue was separated to obtain the subcutaneous tumor. Liquid chromatography with tandem mass spectrometry (LC-MS/MS) was performed using an Agilent 1290 series HPLC system (Agilent Technologies, Palo Alto, CA, USA) coupled to an Agilent 6460 triple-quadrupole mass spectrometer (Agilent Technologies, Palo Alto, CA, USA) equipped with electrospray ionization (ESI). Analytes were separated with matrices using a Waters Symmetry 300 C18 column (2.1 × 100 mm, 3.5 μm; Torrance, CA, USA) maintained at 20 °C. The mobile phase of the pemetrexed group samples was 1% formic acid and 0.5% methanol, and the flow rate was 0.3 ml/min. The mobile phases of the gemcitabine and paclitaxel group samples were 10 mM ammonium acetate in aqueous solution and acetonitrile, and the flow rates were 0.4 ml/min and 0.45 ml/min, respectively. A triple-quadrupole mass spectrometer was used to detect the analytes by ESI in the positive mode. MRM mode was used to detect the product ions. The MS parameters were as follows: pemetrexed group samples: Gas Temp: 350 °C, Gas Flow: 10 l/min, Nebulizer: 40 psi, Sheath Gas Heater: 350 °C, Sheath Gas Flow: 9 l/min, and Capillary: 4000; gemcitabine group samples: Gas Temp: 350 °C, Gas Flow: 8 l/min, Nebulizer: 40 psi, and Capillary: 4000; and paclitaxel group samples: Gas Temp: 350 °C, Gas Flow: 10 l/min, Nebulizer: 40 psi, Sheath Gas Heater: 350 °C, Sheath Gas Flow: 8 l/min, and Capillary: 3500. The column effluent was monitored at the following precursor–product ion transitions: m/z 428.1 → 163.1 for pemetrexed, m/z 641.1→112.0 for gemcitabine and m/z 876.3 → 308.0 for paclitaxel, with a dwell time of 100 ms for each ion transition. The total run time was 6 min for pemetrexed, 8 min for gemcitabine and 5 min for paclitaxel.

### Statistical analysis

All experiments were performed at least three times. Data are expressed as the mean ± SEM. All statistical analyses were performed using one-way analysis of variance (ANOVA) followed by a Dunnett post hoc test with Prism 6.00 software (GraphPad Software, San Diego, CA, USA) and SPSS version 20 (SPSS Inc., Chicago, IL, USA). P < 0.05 was considered to indicate significant differences.

## Results

### Tumor development after stereotactic intracranial injection of tumor cells

**Fig. 1:**
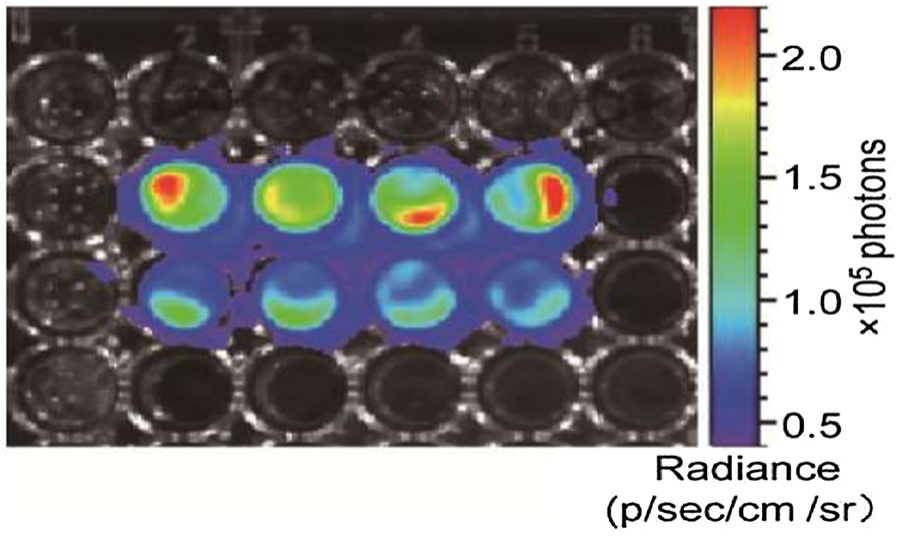
The cell fluorescence intensity of the Lewis cells transfected with the Luc reporter gene. The fluorescence intensity of single cell reached more than 102 p/sec/cm^2^/Sr, indicating the cells were transfected successfully for subsequent experiments

**Fig. 2:**
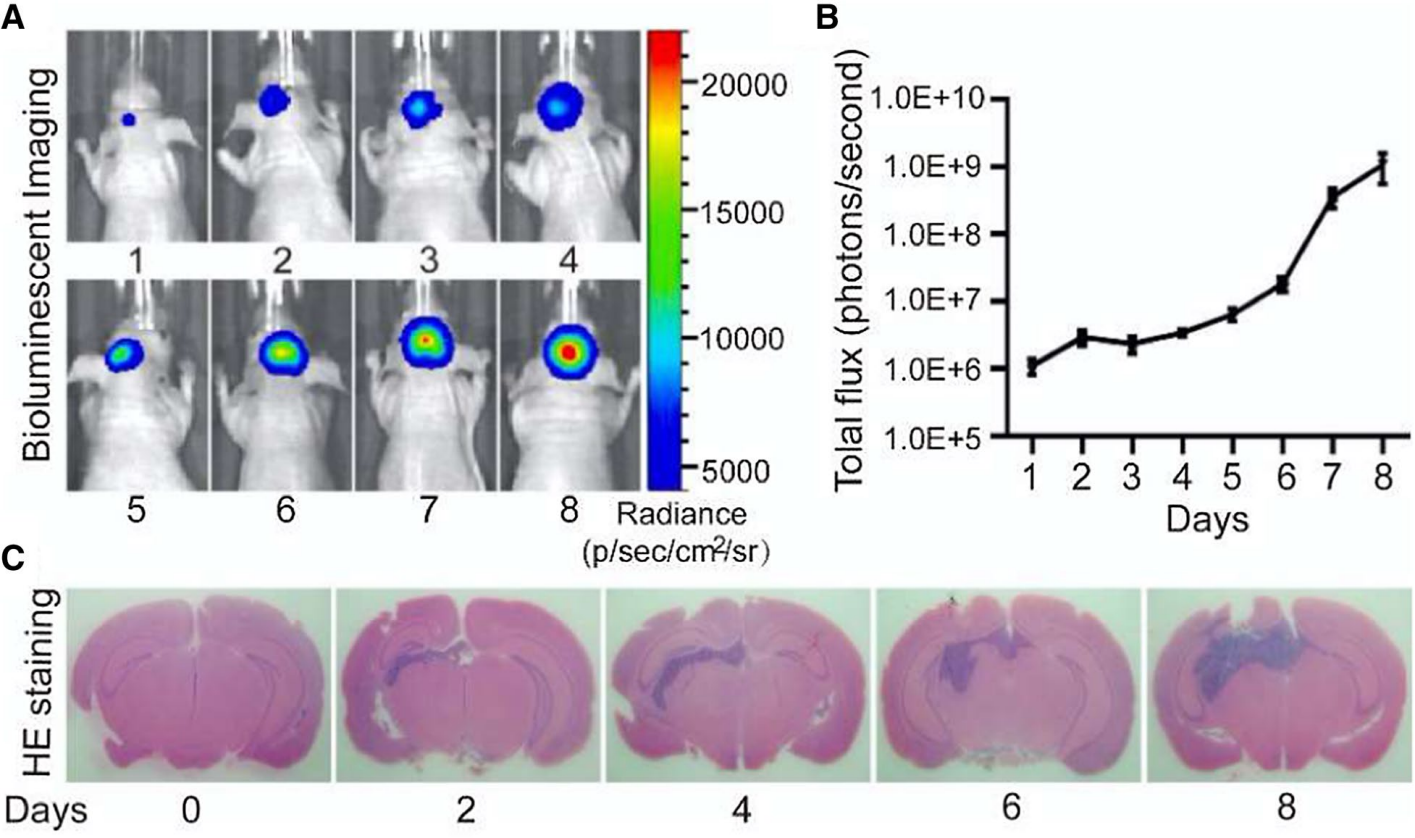
**A** and **B** Tumor fluorescence intensity detected by a small animal imaging system. **C** H&E staining of brain sections of tumor-bearing mice. Data are expressed as x ± SEM, n=5

Lewis cells were transfected with Luc reporter gene. Cells with fluorescence intensity above 10^2^ p/sec/cm^2^/sr were successfully transfected and were used in subsequent experiments (Fig. 1). From the 7th day after injection, some mice exhibited activity disorder, seizures and convulsions. As shown in Fig. 2, the volume of brain metastases increased gradually after implantation of Lewis cells measured by fluorescence intensity and H&E staining.

### BTB permeability

**Fig. 3:**
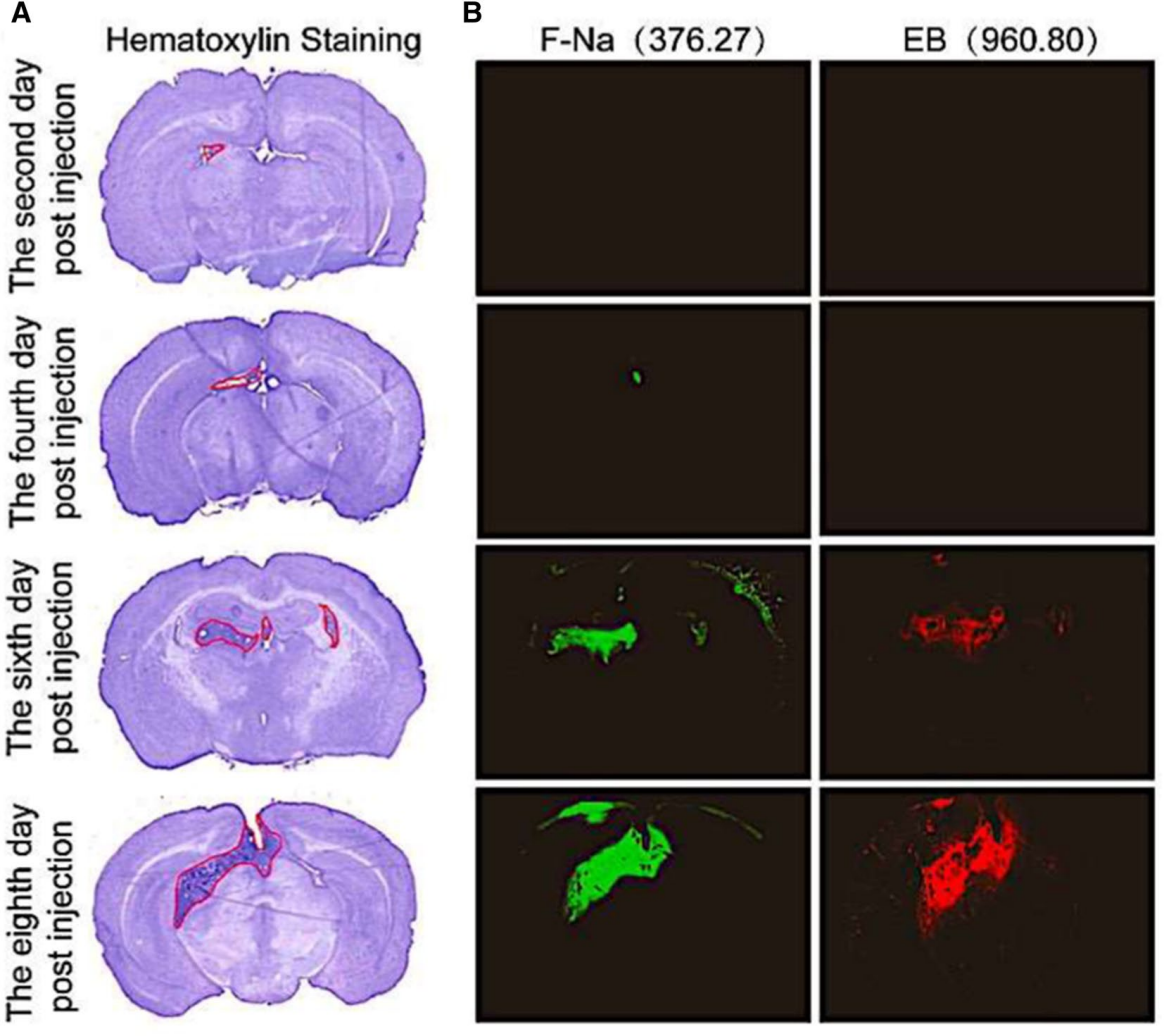
**A** Hematoxylin staining of mice brain at different time points after implantation of Lewis cells. **B** The permeability of two fluorescent dyes at different time points after implantation of Lewis cells. Green fluorescence dye indicates fluorescein sodium (F-Na), and its molecular weight is 376.27. Red fluorescence dye indicates evans blue (EB), the molecular weight is 960.80. The fluorescence intensity increased over time after implantation of Lewis cells. F-Na and EB penetrated the brain from the fourth and sixth day after injection of cells, respectively

Considering that the permeability of BTB may be related to tumor size, we selected two kinds of fluorescent dyes with different molecular weight to dynamically observe the permeability of BTB of brain metastases. As shown in Fig. 3B, the fluorescence of F-Na (the low molecular weight tracer) was observed in brain metastases from the fourth day after injection of Lewis cells, while both the high and low molecular weight tracers (EB and F-Na) penetrated the metastases from the sixth day after injection. However, no fluorescence was observed in normal area of the brain. The results showed that the permeability of BTB in metastatic lesions was increased compared with normal BBB. Additionally, the permeability of BTB increased with the time of cells injection, which was related to tumor size.

### Structural differences between BBB and BTB

**Fig. 4:**
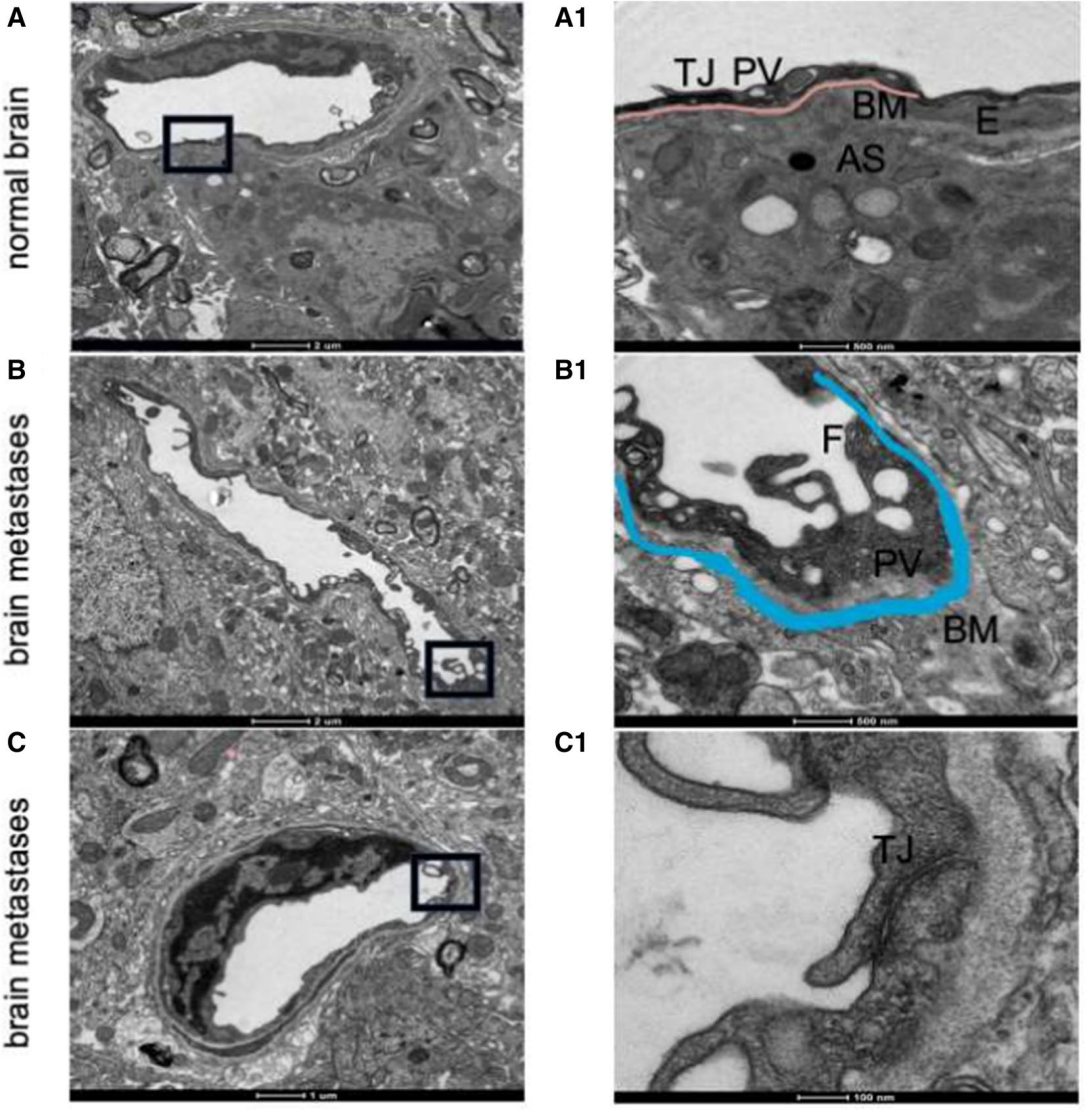
The ultrastructure of the BBB and BTB in brain metastases. The normal brain of figure **A** was obtained by the brain tissue of health mouse without injection of tumor cells. The brain metastases were observed on the sixth day after injection of tumor cells. **A1**–**C1** represent enlarged images of the black frame part in **A**–**C**. *AS* astrocyte, *TJ *tight junction, *BM* basement membrane, *E* endothelial cells, *F* fenestra of the endothelial cell, *PV* pinocytotic vesicle. The pink line in A1 represents the basement membrane of BBB in normal brain. The blue line in B1 represents the basement membrane of BTB in brain metastases

**Fig. 5:**
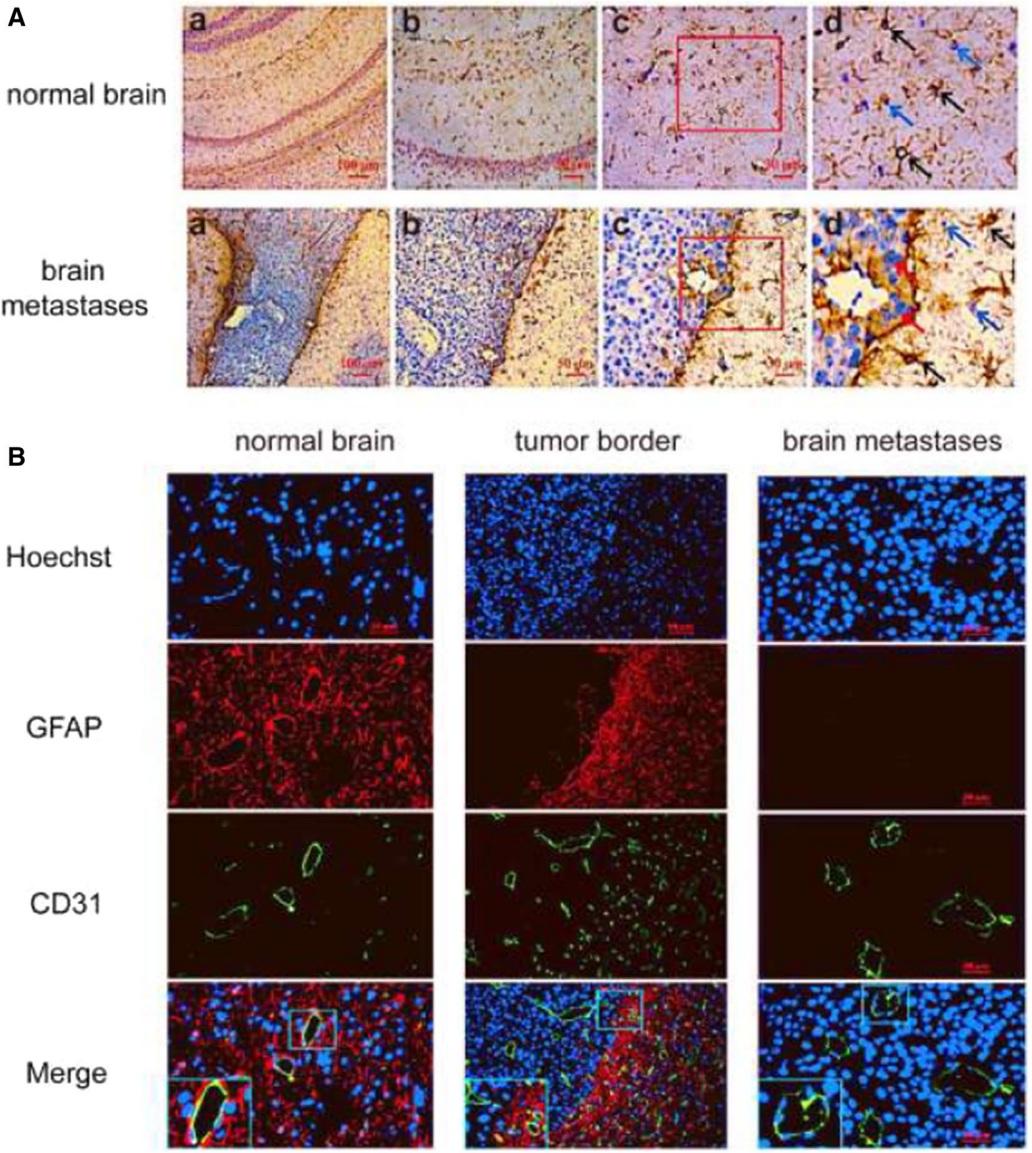
**A** The immunohistochemistry (IHC) of brain tissue sections of mice. The black arrows represent astrocyte, the blue arrows indicate brain tissue, and the red arrows indicate the tumor. Figure **d** is an enlarged portion of the red frame in Figure **c**. **B** The immunofluorescence (IF) of brain tissue sections of mice. The nuclei were stained by Hoechst. GFAP and CD31 were specific markers of astrocytes and endothelial cells, respectively. The normal brains in figure **A** and **B** were obtained by the brain tissues of health mice without injection of tumor cells, the brain metastases were observed on the sixth day after injection of tumor cells

The increased permeability of BTB in brain metastases suggests that the BBB structure may be damaged. To further study the structural differences between BTB and normal BBB, we observed their ultrastructure by TEM. In Fig. 4, A and A1 showed the normal BBB, while B, B1, C and C1 were the BTB of brain metastases. As shown in Fig. 4B1, thickened basement membranes, increased fenestrae, and pinocytotic vesicles were found in the BTB of brain metastases. In Fig. 4C1, the tight junction between endothelial cells were opened. The IHC and IF staining showed that astrocytes were distributed surrounded the blood vessels both in normal brain and at the tumor border, but no astrocytes were found in brain metastases (Fig. 5).

### Effects of tumor size on BBB structure

**Fig. 6:**
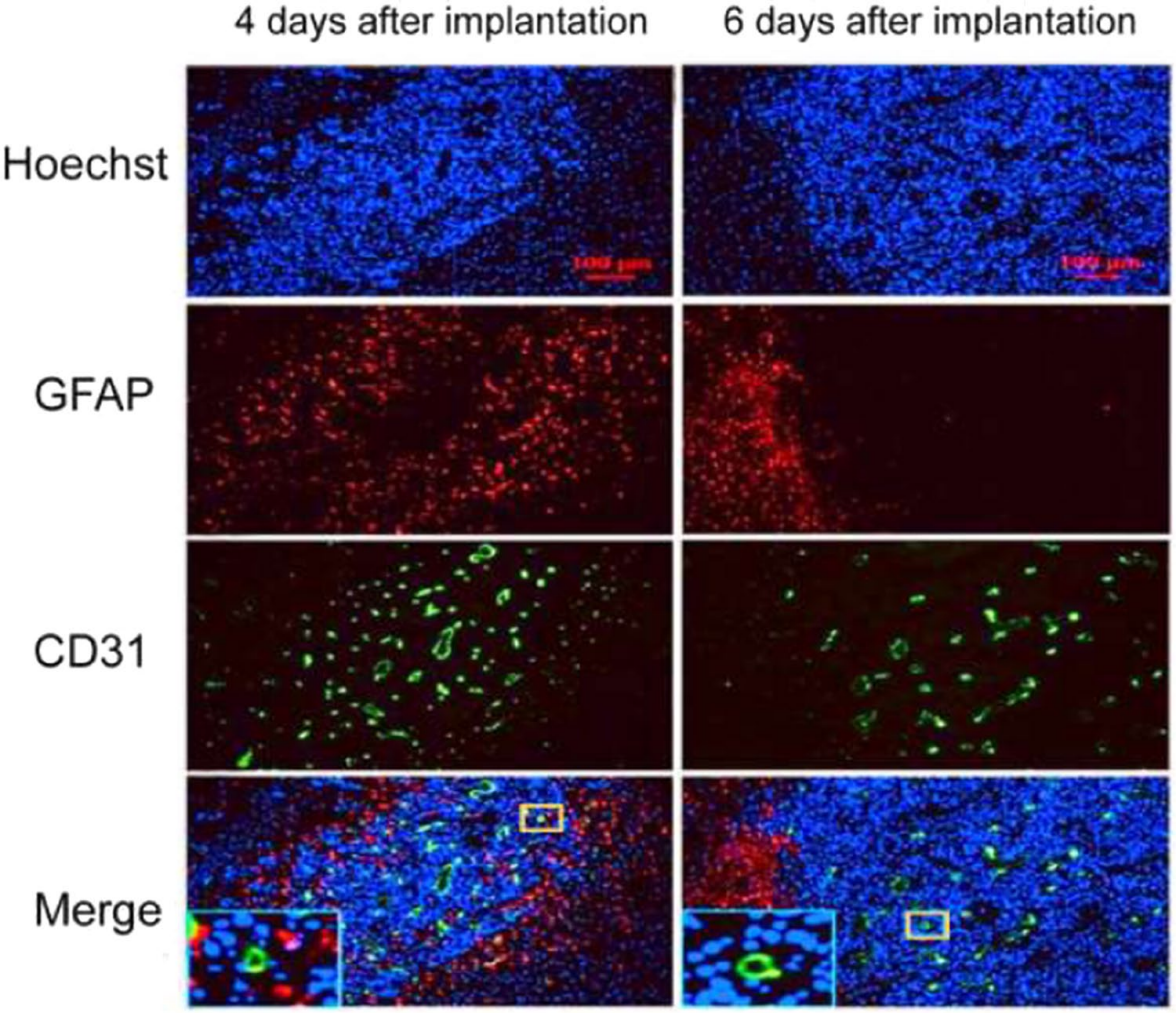
The immunofluorescence (IF) of brain tissues at different time points after implantation of cells. The nuclei were stained by Hoechst. GFAP and CD31 were specific markers of astrocytes and endothelial cells, respectively. The lower left rectangular boxes in figure Merge were the enlarged portion of the yellow frame in the figures

Figure 6 shows that most astrocytes were recruited at the border of brain metastases, while there are scattered astrocytes migrating around the blood vessels within the relatively small tumor foci after 4 days of implantation. As the tumor grows (after 6 days of implantation), the numbers of astrocytes within the brain metastases decreased significantly and disappeared ultimately. However, astrocytes were still visible at the boundary of brain metastases.

### Distribution of chemotherapeutic drugs

**Fig. 7:**
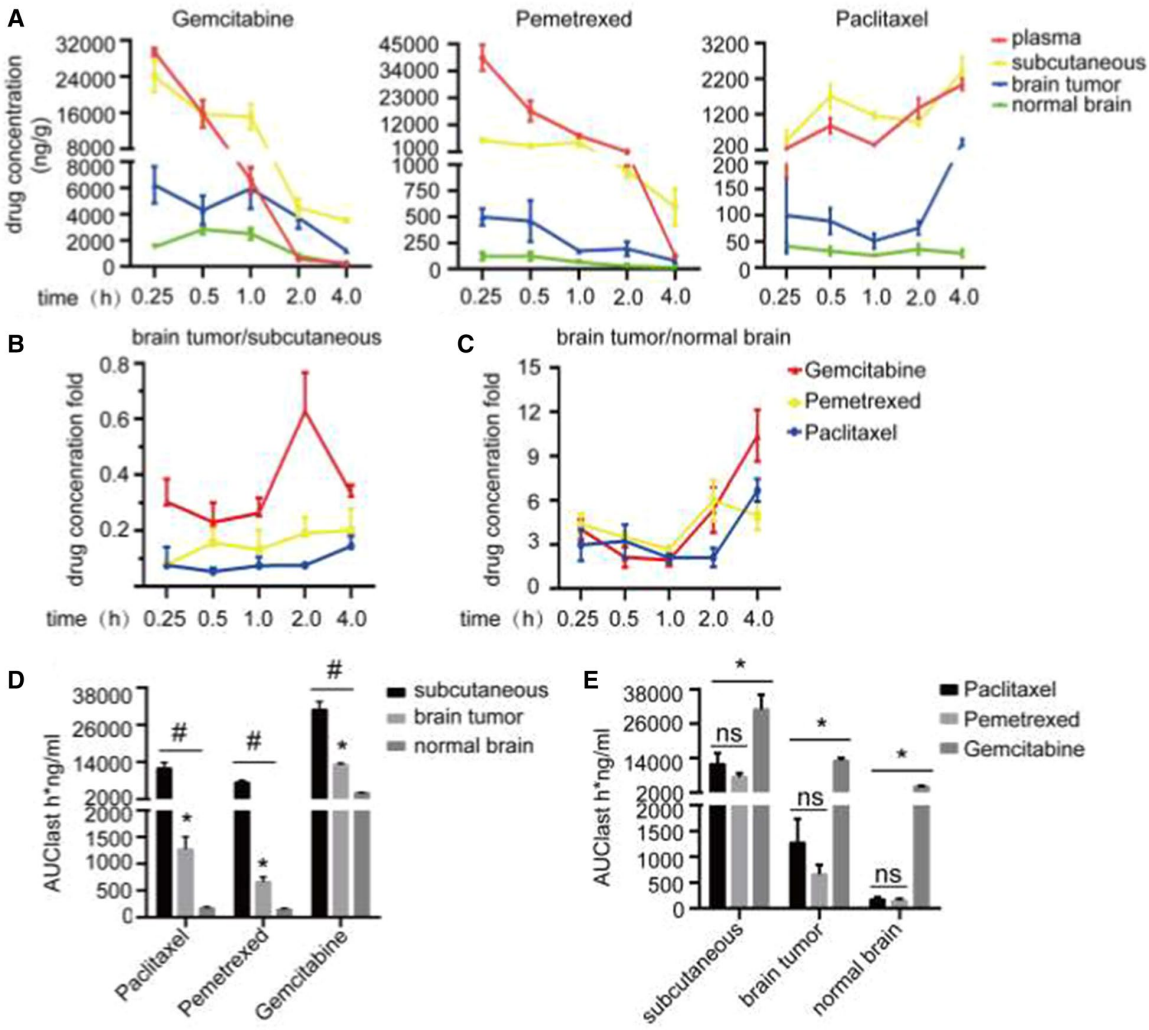
LC-MS/MS was used to detect the distribution of chemotherapeutic drugs in different tissues on the sixth day after injection of tumor cells. **A** The concentrations of the chemotherapeutic drugs in each tissue at different time points after treatment. **B** The ratio of the concentrations of various drugs in brain metastases to that in subcutaneous tissues. **C** The ratio of the concentrations of the three drugs in brain metastases to that in normal brain. **D** The area under the concentration-time curve of three chemotherapeutic drugs (AUClast), showing the distribution of each drug in different tissues. ^#^P<0.05, versus subcutaneous tumors, *P<0.05 versus normal brain. **E** The area under the concentration-time curve of the drugs (AUClast), showing the distribution of the three different drugs in each tissue. *P<0.05 versus gemcitabine. (x ± SEM, n=4)

The distribution of three chemotherapeutic drugs (gemcitabine, paclitaxel and pemetrexed) in each tissue was detected by LC-MS/MS. Figure 7A shows the concentrations of the three chemotherapeutic drugs in each tissue at different time points after treatment. It could be seen the concentrations of gemcitabine and pemetrexed in various tissues gradually decreased within 0.25 to 4 h, indicating that the drugs were quickly distributed and metabolized rapidly. The concentrations of paclitaxel in the tissues were still rising 4 h after injection, suggesting that its absorption, distribution and metabolism were relatively slow, and its half-life was long. In addition, we could see that the concentrations of these drugs in brain metastases were higher than those in normal brain tissues. Figure 7B was the ratio of the concentrations of various drugs in brain metastases to that in subcutaneous tissues. We found the concentrations of the three drugs in brain metastases were lower than those in subcutaneous tissues. From Fig. 7C, we could see that the concentrations of the drugs in brain metastases were higher than those in normal brain tissues. Figure 7D was the area under the drug concentration time curve (AUClast), showing the distribution of each drug in different tissues (subcutaneous tumors, brain metastases and normal brain tissues). It could be seen that the distribution of these three drugs in subcutaneous tumors were higher than that in brain metastases, and the distribution in normal brain tissues were the lowest. Figure 7E was the distribution of these three different drugs in the three tissues. The concentration of gemcitabine was highest in each tissue, while there was no significant difference between the concentrations of pemetrexed and paclitaxel.

## Discussion

Lung cancer remains the leading cause of cancer-related mortality worldwide [18]. The propensity for metastasis to the central nervous system (CNS) is a major problem in the management of lung cancer patients. It usually represents a poor prognosis since most patients are at the advanced stage of the disease progression [19]. Thus, it is important to explore the brain metastases of NSCLC. The dismal prognosis of lung cancer brain metastasis involves in many mechanisms, BBB is one of the most important factors.

Under physiological conditions, BBB is regarded as a neurovascular unit, composing of tightly connected endothelial cells and maintained by crosstalk with astrocytes and pericytes. BBB plays a vital role in restricting the passage of circulating macromolecules into brain parenchyma [20]. The BBB and the lack of a lymphatic system are responsible for maintaining the brain as an immunologically privileged site. In addition, BBB protects the brain against the entry of microorganisms and most drugs, maintaining the stability of the brain environment [21]. Recently, increasing evidence have shown that when brain metastases occur, the formation of BTB replaces the normal BBB, exhibiting different structural and functional characteristics. The protective effects of the BTB are significantly weakened [22]. Therefore, a mice model of NSCLC metastasis in brain was established to fully elucidate the permeability and ultrastructure between BBB and BTB.

Our study showed that the BBB was disrupted in brain metastases of lung cancer, and the permeability was increased as the tumor growth. The BBB permeability reveals a significant size effect in brain metastases. Previous studies have shown that the BBB permeability of brain metastases larger than 0.5 mm in diameter is significantly increased compared to that of scattered infiltrating and smaller lesions. With an increase in tumor volume, ischemic necrosis may occur in the center of the lesion, promoting the synthesis and secretion of vascular endothelial growth factor (VEGF) [23, 24]. VEGF can increase BBB permeability via destroying VE-cadherin-β-catenin junction complex [25]. In addition, the up-regulation in endothelial cell vesicle density through up-regulation of pit protein expression is another important mechanism for increased BBB permeability [26]. Despite of much effort, more in-depth studies are still needed to further understand these complex mechanisms.

From the ultrastructure results, we could see that the structures of the barriers in metastases changed substantially, and blood vessels at the invasive edge and center of the tumors were abnormal. Compared with those in healthy brain tissue, in the metastatic lesions, the endothelial cells were damaged, the basement membranes were thicker, the endothelial tight junctions were open, the numbers of fenestrae and pinocytotic vesicles were increased, and astrocyte encapsulation was absent around blood vessels. However, at the edge of the tumor, the barrier structure was intact, indicating that such damage was heterogeneous, and these structural differences were related to tumor size. One of the interesting findings in our study was that astrocytes were recruited at the border of brain metastases and within the relatively small tumor foci, but they were disappeared within the big brain metastases.

Astrocytes, as important components of the BBB, participate in neurogenesis, neuronal proliferation, migration, differentiation, neuronal signal transduction, nutrient transport, secretion of neurotrophic factors, and immune activation to prevent the invasion of foreign substances. Therefore, astrocytes have been considered as key orchestrators for regulating BBB integrity and permeability [27, 28]. Previous studies have suggested that early contact between tumor cells and astrocytes will lead to tumor cell death and remove most tumor cells entering the brain. To successfully settle in the brain, tumor cells generated characteristics of preventing the pro-apoptotic stimulation induced by astrocytes [29]. However, many studies have shown that astrocytes could promote the growth and progression of established brain metastases [30]. What’s more, in a study of oligodendroglioma, tumor cells in the brain could shed micro vesicles to induce astrocytes death [31]. Therefore, there is also complex communication between tumor cells and astrocytes in the tumor microenvironment of brain metastases, which still needs to be further explored.

As one of the commonly used clinical treatment schemes, chemotherapy is essential in the systemic treatment of brain metastases. Therefore, it is particularly important to formulate a reasonable individualized scheme according to the patient’s physical condition, pathological type and intracranial lesions [32]. However, for a long time, the effective rate of chemotherapy in patients with brain metastasis is very low. The traditional concept attributed this to the difficulty of drugs penetrating the BBB. Studies have found that due to the lack of lymphatic system, astrocytes serve as immune modulator preventing the invasion of microbial and the entry of drugs [21, 33]. In addition, the transport proteins distributed on the membrane of brain capillary endothelial cells also play important roles in maintaining the selective permeability of the BBB [34]. P-gp, an ATP-dependent phosphoglycoprotein, is highly expressed in endothelial cells of BBB, and this high expression of P-gp is found to be an important mechanism of multidrug resistance (MDR) in tumors. Animal studies have shown that P-gp inhibitors can increase the concentration of chemotherapeutic drugs in brain metastases, improving the chemotherapeutic efficiency of brain metastases from lung cancer [35]. Our study found that although the BBB in brain metastases was destroyed and the permeability increased, the concentrations of chemotherapeutic drugs in brain metastases was higher than that in normal brain tissues, but they were still lower than that in subcutaneous tumors. This suggested that BTB in brain metastases could still prevent part of chemotherapeutic drugs from penetrating into the lesions.

Besides, the infiltration of chemotherapeutic drugs into the brain is not only related to the BBB structure, but also to the molecular size, charge state, liposolubility and binding power with plasma proteins of the drug. Drugs with a low molecular weight, low plasma protein binding rate and high liposolubility can easily penetrate the BBB, while water-soluble drugs with high molecular weight and polarity have difficulty in penetrating the BBB. Among the three chemotherapeutic drugs in our study, gemcitabine had the smallest molecular weight, followed by pemetrexed, paclitaxel had the largest molecular weight, but only paclitaxel was lipophilic. We found that gemcitabine had the highest concentration in brain metastases, probably because of its small molecular weight. The molecular weight of paclitaxel was larger than that of pemetrexed, but its concentration in brain metastases was slightly higher than that of pemetrexed, which may be due to its high liposolubility, suggesting that the permeability of chemotherapeutic drugs in brain metastases is closely related to their molecular weight and liposolubility.

## Conclusion

The present work demonstrated that NSCLC metastases in the brain disrupt the BBB leading to the formation of BTB, and this disruption was not homogeneous. The permeability of the impaired barrier was associated with the tumor size. Chemotherapeutic agents can pass through the BTB of metastatic lesions but have difficulty in penetrating the normal BBB. The permeability of chemotherapeutic agents is related to their molecular weight and liposolubility. Understanding the structural differences between BBB and BTB shed light on improving chemotherapeutic drug transport for treating brain metastases in NSCLC.

## Data Availability

The data supporting the conclusions of this study are included within the article.

## Abbreviations

NSCLC: Non-small cell lung cancer
BBB: Blood–brain barrier
BTB: Blood–tumor barrier
TEM: Transmission electron microscopy
SRS: Stereotactic radiosurgery
WBRT: Whole-brain radiotherapy
EB: Evans blue
F-Na: Fluorescein sodium
H&E: Hematoxylin and eosin
IHC: Immunohistochemistry
IF: Immunofluorescence
DAB: Diaminobenzidine
ESI: Electrospray ionization
LC-MS/MS: Liquid chromatography with tandem mass spectrometry
ANOVA: One-way analysis of variance
CNS: Central nervous system
MDR: Multidrug resistance
VEGF: Vascular endothelial growth factor

## Materials availability

The data used in the current study are available from the corresponding author on reasonable request.
